# Merkel cell carcinoma in a patient on ruxolitinib treatment for polycythemia vera

**DOI:** 10.1016/j.jdcr.2026.06.071

**Published:** 2026-07-20

**Authors:** Suraj Patel, Milena Dragovic, Anja Olsen, Brett Bender

**Affiliations:** a Department of Dermatology, Corewell Health Farmington Hills Hospital, Farmington Hills, Michigan; b Lincoln Memorial University DeBusk College of Osteopathic Medicine, Harrogate, Tennessee; c Clarkston Dermatology Group, Clarkston, Michigan

**Keywords:** Janus kinase inhibitor, Merkel cell carcinoma, polycythemia vera, surveillance

## Introduction

Merkel cell carcinoma (MCC) is a rare aggressive neuroendocrine skin cancer derived from Merkel cells located in the basal layer of the epidermis and follicular epithelium. MCC predominantly affects elderly patients, particularly white males, and results from a combination of risk factors including genetic, molecular, age-related-immune decline, and environmental factors.^1^ Long-term ultraviolet radiation is a key risk factor for MCC, particularly for tumors arising on sun-exposed areas such as the head and neck, although less advanced lesions at diagnosis are more often seen on the limbs.^1^ MCC is also correlated with involvement of a viral pathogen, Merkel cell polyomavirus, and can be associated with iatrogenic immunosuppressive medications, emphasizing the role of immune dysfunctions in MCC pathogenesis.^2^

MCC lesions present as rapidly growing painless nodules or hardened plaques with a red or purple color; however, this is relatively nonspecific, which may lead to a delay in diagnosis.^1^ Histopathologically, MCC is characterized by a trabecular growth pattern in the dermis with small, round, blue cells containing dense-core granules located peripherally within the cytoplasm.^3^ Diagnosis relies on immunohistochemical staining, cytokeratin-20, highlighting the distinct dot-like pattern.^1^ MCC is clinically aggressive and displays a high mortality rate due to a high tendency for local recurrence, regional lymph node metastasis, and distant dissemination.^4^ Despite advances in understanding MCC, the mechanisms of invasion and metastasis remain incompletely defined, emphasizing the need for continued research to improve diagnostic accuracy, therapeutic strategies, and patient outcomes.

## Case report

We present the case of an 80-year-old female seen in our dermatology clinic for evaluation of a skin lesion located on the left anterior thigh (Fig 1). The patient's medical history was significant for hypercholesterolemia, hypertension, nicotine dependence, arthritis, polycythemia vera, and pulmonary embolism. She was diagnosed with breast cancer in 2007 and treated with lumpectomy and radiation. In 2011, the patient was diagnosed with polycythemia vera and started on ruxolitinib and hydroxyurea. The patient discontinued hydroxyurea in 2016 due to gastrointestinal side effects but continued ruxolitinib treatment. In 2023, she was diagnosed with small cell lung cancer and treated with radiation therapy. The patient denied any family or personal history of skin cancer. Her medications at presentation included lisinopril, rivaroxaban, oral ruxolitinib, risperidone, and rosuvastatin.

**Fig 1:**
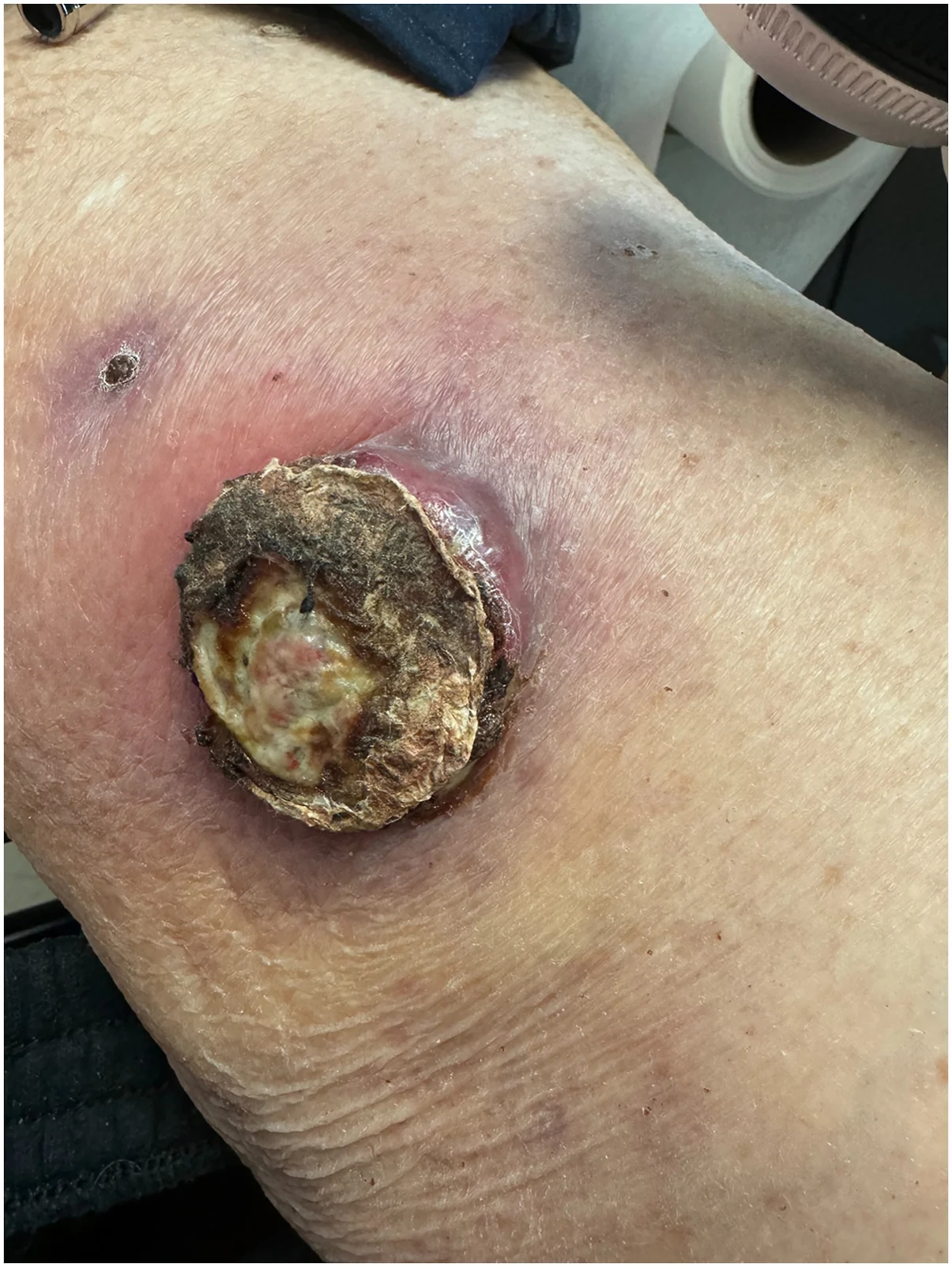
Clinical presentation. On the left anterior proximal thigh, a 5.5 × 4.5 cm crusted plaque with an ulcerated center and an erythematous to violaceous rim.

The patient endorsed that the left anterior thigh lesion had been present for approximately 3 to 4 months and grew rapidly since onset. At presentation, the lesion measured 5.5 × 4.5 cm. She complained the lesion was itchy, often irritated by clothing, and bled intermittently. A shave biopsy was performed along the lateral margin of the lesion. The biopsy specimen measured 0.9 × 0.6 × 0.2 cm and pathology was consistent with MCC extending to the deep margins (Fig 2). The specimen stained positive for CK AEl/AE3, synaptophysin, and chromogranin. Cytokeratin-20 and thyroid transcription factor 1 were largely negative. Thyroid transcription factor 1 negativity is a core confirmatory feature used to diagnose MCC and distinguish from small cell lung cancer and other neuroendocrine carcinomas.^5^ These findings were consistent with MCC (Fig 3). The patient's lesion continued to rapidly grow larger after biopsy. The patient underwent inpatient surgical excision which resulted in a large defect that was left to heal by secondary intent with a biograft (Fig 4).

**Fig 2:**
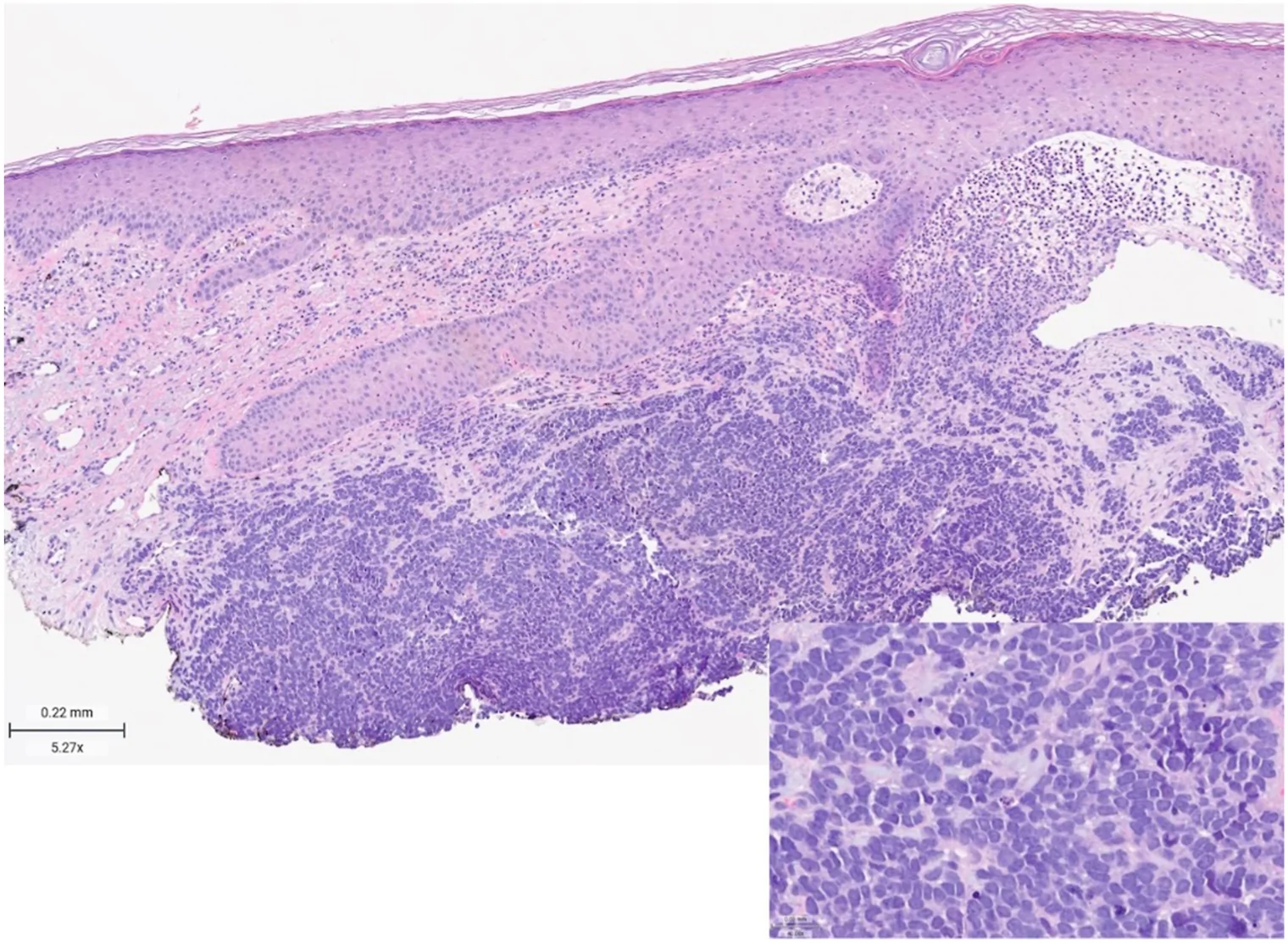
(H&E 5× with inset at 40×). Dermal infiltrate composed of sheets of uniform basaloid cells with high nuclear: cytoplasm ratio (inset). Neuroendocrine carcinoma consistent with Merkel cell carcinoma involving the deep margin.

**Fig 3:**
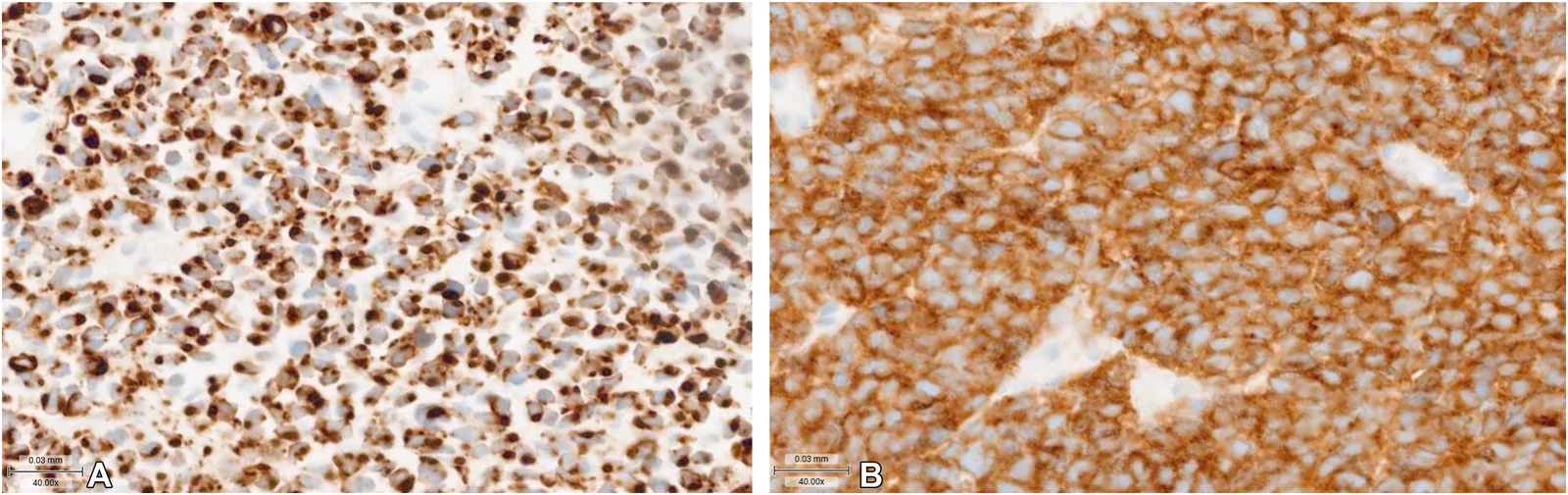
**A,** (40×) By immunohistochemistry, lesional cells are strongly and diffusely positive for CK AEl/AE3 in paranuclear dot-like pattern. **B,** Synaptophysin with cytoplasmic dot-like accentuation.

**Fig 4:**
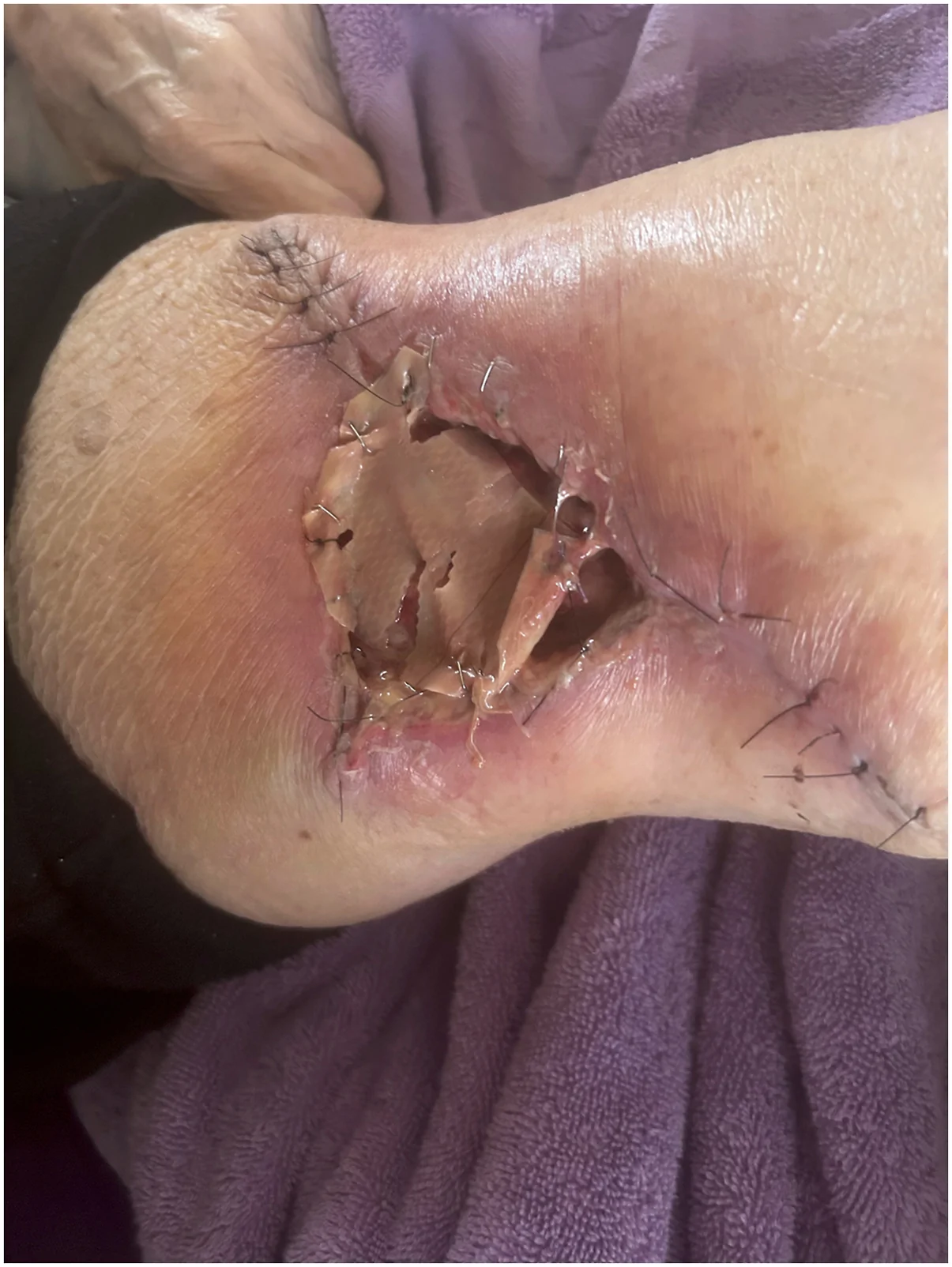
Two weeks status postwide local excision. The surgical defect was partially closed using primary intent, with the residual central defect left to close by secondary intent with the aid of a biograft.

The patient was referred to oncology for further management. A positron emission tomography-computed tomography scan was performed to evaluate disease extent for staging purposes and revealed 2 positive lymph nodes in the left femoral and periaortic lymph node basins. She was diagnosed with stage 4 MCC and started on IV pembrolizumab 200 mg every 3 weeks. She was restarted on hydroxyurea 500 mg orally twice daily for PV and ruxolitinib was discontinued by oncology due to immunosuppression. After 2 cycles of pembrolizumab, patient could not tolerate gastrointestinal side effects and elected against any further treatment. Pembrolizumab was discontinued and testing for Merkel cell polyomavirus was not performed. The patient elected comfort care and passed away 4 months later.

## Discussion

Janus kinase inhibitors (JAKis) are medications used in treatment of myeloproliferative neoplasms such as polycythemia vera. Our patient was on therapy with oral ruxolitinib, a potent selective inhibitor of JAK1 and JAK2, for over 10 years for treatment of polycythemia vera. Ruxolitinib can improve the quality of life of patients with myeloproliferative neoplasms by reducing splenomegaly and symptom burden. However, clinical trials have found that treatment with JAKis for over 5 years has been identified as a possible contributor to NMSCs in several case reports.^6^^,^^7^ One case reported 2 synchronous, fast growing lesions composed of Merkel cell carcinoma (MCC) in collision with squamous cell carcinoma and basal cell carcinoma. This collision tumor occurred in an 80-year-old male patient with a V617F JAK2-positive myeloproliferative neoplasm after 6 years of treatment with ruxolitinib.^6^ Another case reported a 47-year-old female patient with a 14-year history of chronic myeloproliferative syndrome that developed MCC after treatment with ruxolitinib for 7 years.^7^

By inhibiting the JAK-STAT pathway, JAKis diminish proinflammatory interleukin and interferon secretion. This weakens cytotoxic T-cell activation and function. While this is useful against some types of malignancies, it can create an environment for malignant cell development into NMSCs.^8^ In this case, the rapidly growing MCC with onset after 14 years of immunosuppressive therapy with ruxolitinib, without any other history of skin cancer or medications, suggests the long-term use of ruxolitinib played a role in development of this MCC.

These cases highlight the association between the use of JAKis and the development of NMSC when treating myeloproliferative disorders. We emphasize the importance of close dermatologic monitoring of these patients with full body skin exams to increase surveillance and detection of MCC and other NMSCs. We recommend close collaboration between oncologists and dermatologists. Patients initiated on treatment with ruxolitinib should be referred to dermatology for routine skin exams at least biannually to facilitate prompt diagnosis of skin cancer in effort to reduce morbidity and/or mortality.

## Conflicts of interest

None disclosed.
